# Clinical Outcomes After Failed Endoscopic Detorsion for Sigmoid Volvulus: A Single‐Center Retrospective Cohort Study

**DOI:** 10.1002/deo2.70368

**Published:** 2026-07-01

**Authors:** Hidehiro Kamezaki, Arisa Kato, Nana Yamada, Terunao Iwanaga, Takafumi Sakuma, Koji Takahashi, Junichi Senoo, Sadahisa Ogasawara, Tomoaki Matsumura, Jun Kato

**Affiliations:** ^1^ Department of Gastroenterology Eastern Chiba Medical Center Chiba Japan; ^2^ Department of General Medical Science Graduate School of Medicine Chiba University Chiba Japan; ^3^ Department of Gastroenterology Graduate School of Medicine Chiba University Chiba Japan

**Keywords:** colonoscopy, endoscopic decompression, endoscopic detorsion, recurrence, sigmoid volvulus

## Abstract

**Objectives:**

Endoscopic detorsion is the first‐line treatment for sigmoid volvulus (SV); however, detorsion is not always successful. We evaluated clinical outcomes after failed endoscopic detorsion and assessed recurrence in patients who achieved clinical success after decompression.

**Methods:**

This single‐center retrospective cohort study included 47 patients with first‐episode SV, of whom 43 underwent endoscopic management. Baseline characteristics and short‐term outcomes were compared between successful and failed detorsion groups. Among patients with clinical success, the in‐hospital course and 1‐year cumulative recurrence were compared between the successful detorsion and successful decompression groups. Cumulative recurrence was evaluated using the Kaplan–Meier method.

**Results:**

Successful detorsion was achieved in 21 of 43 patients (48.8%). Among 22 patients with failed detorsion, 17 achieved clinical success after decompression, whereas five had clinical failure, including four who underwent emergency surgery and one who died the following day. The overall clinical success rate was 88.4% (38/43). Failed detorsion was associated with lower room‐air oxygen saturation and higher lactate levels. Clinical success was less frequent in the failed detorsion group (77.3% vs. 100%, *p* = 0.065), and hospital stay was longer (median 13 vs. 7 days, *p* = 0.071). Among patients with clinical success, time to resumption of oral intake was shorter in the successful detorsion group (median 1.5 vs. 3 days, *p* = 0.004), whereas the 1‐year cumulative recurrence rate was numerically higher but not statistically significant (79.6 vs. 51.3%, log‐rank *p* = 0.087).

**Conclusions:**

After assessing the need for emergency surgery, decompression alone may not always require immediate repeat detorsion in selected patients who improve clinically.

**Trial Registration:**

N/A.

## 1 Introduction

Sigmoid volvulus (SV) is a large‐bowel obstruction caused by twisting of the sigmoid colon around its mesenteric axis. Progressive torsion can cause obstruction, ischemia, necrosis, and perforation; therefore, timely intervention is essential [1, 2]. SV remains clinically relevant in Japan, especially among older adults and patients with constipation, neuropsychiatric disorders, impaired mobility, or long‐term care facility residence [1, 2, 3, 4].

In the absence of peritonitis, ischemia, or perforation, urgent endoscopic management is widely accepted as first‐line treatment because it is less invasive and rapidly relieves obstruction [2, 5, 6]. Endoscopic detorsion is generally preferred because it relieves obstruction and corrects torsion. However, detorsion is not always technically successful, and recurrence after initial endoscopic treatment is common [4, 7, 8]. Guidelines recommend elective sigmoidectomy after successful endoscopic reduction in appropriate surgical candidates; however, many patients are older or frail and are managed nonoperatively in routine practice [2, 5, 6].

When complete detorsion cannot be achieved or further attempts are deemed unsafe because of prolonged procedure time, patient frailty, or poor maneuverability, endoscopists may perform decompression alone to reduce colonic distension and relieve symptoms. Some patients show sufficient improvement to resume oral intake without immediate surgery; however, the clinical significance of this improvement remains uncertain. Evidence is limited, particularly regarding the short‐term course and recurrence patterns among patients who achieve clinical success after decompression alone. Therefore, we conducted a single‐center retrospective cohort study to compare short‐term outcomes after successful versus failed detorsion and to assess in‐hospital course and cumulative recurrence among patients who achieved clinical success after decompression.

## 2 Methods

### 2.1 Study Design and Setting

This retrospective, single‐center cohort study was conducted at Eastern Chiba Medical Center. Consecutive patients with first‐episode SV treated between April 2014 and May 2025 were included. Electronic medical records and endoscopy reports were reviewed through December 31, 2025, to ascertain clinical outcomes following the index episode.

### 2.2 Participants and Group Classification

During the study period, 47 patients with first‐episode SV were identified. Four who underwent initial emergency surgery were excluded, leaving 43 endoscopically managed patients. For baseline and short‐term outcome analyses, patients were classified into successful and failed detorsion groups. Failed‐detorsion patients with clinical success after decompression were classified as the successful decompression group; the remainder were classified as the clinical failure group.

### 2.3 Diagnosis and Definitions

SV was diagnosed from clinical presentation and radiologic findings, including abdominal computed tomography (CT) and/or radiography, and confirmed endoscopically when applicable. Uncomplicated SV was defined as SV without evidence of peritonitis, bowel ischemia, or perforation [2, 5, 6, 9]. Successful detorsion was defined as endoscopic release of torsion with effective decompression, confirmed by endoscopic and fluoroscopic findings, including straightening and shortening of the colonoscope. Postprocedural CT or radiography was not used to define successful detorsion. Failed detorsion and decompression alone were defined as incomplete release of torsion despite an attempt, with endoscopic aspiration of intraluminal gas when further manipulation was unsafe [2, 5, 6, 9]. Clinical success was defined as resumption of oral intake during the index hospitalization without emergency surgery after endoscopic management. Clinical failure was defined as emergency surgery after endoscopy or death during the index hospitalization without clinical improvement after decompression.

### 2.4 Endoscopic Management

Urgent colonoscopy was performed by trained endoscopists using a high‐definition pediatric colonoscope (PCF‐H290L; Olympus Medical Systems Corp., Tokyo, Japan) under fluoroscopic guidance in all cases. CO2 insufflation was used, with insufflation minimized, and repeated aspiration was performed to reduce distension. The colonoscope was gently advanced to the torsion site, and detorsion was attempted using careful torque, advancement, withdrawal, and shortening maneuvers. When the colonoscope could be advanced beyond the torsion, proximal decompression was achieved by aspiration. If complete detorsion was unsuccessful or unsafe, decompression alone was performed [2, 5, 6, 9]. Water‐immersion colonoscopy was not used. Endoscopic mucosal findings were categorized as normal mucosa, erythema, or necrosis.

### 2.5 Data Collection

Data were extracted from electronic medical records and endoscopy reports. Baseline variables included age, sex, body mass index (BMI), and performance status. Performance status was assessed using the Eastern Cooperative Oncology Group (ECOG) Performance Status scale, with scores ranging from 0 to 4 and higher scores indicating greater functional impairment. Comorbidities and background factors included dementia, cerebrovascular disease, Parkinsonism, psychiatric disorders, cardiac disease, respiratory disease, diabetes mellitus, prior abdominal surgery, laxative use, and residence in a long‐term care facility or chronic care hospital. This variable indicated residence in a facility providing long‐term care or ADL assistance, or admission to a chronic care hospital before the index SV admission. Clinical presentation at admission included body temperature, peripheral oxygen saturation on room air (SpO_2_), abdominal distension, and spontaneous abdominal pain. Laboratory variables included serum albumin, lactate dehydrogenase, estimated glomerular filtration rate, creatine kinase, hemoglobin A1c, C‐reactive protein, white blood cell count, platelet count, prothrombin time–international normalized ratio, and lactate.

For patients who underwent initial emergency surgery or urgent surgery after endoscopic management or those who died during the index hospitalization, additional individual‐level data were collected, including management pathway, endoscopic mucosal findings when available, CT findings, including the presence of ascites, lactate level, time from endoscopy to surgery or death, length of hospital stay, and clinical outcome.

### 2.6 Outcomes

The primary short‐term outcomes were clinical success, emergency surgery, 30‐day mortality, and length of hospital stay. Oral intake was resumed after clinical and radiographic improvement, including relief of abdominal distension and/or abdominal pain, absence of peritoneal signs or deterioration, stable vital signs, and, when documented, passage of flatus or stool. Abdominal radiographs were reviewed in all patients before oral intake to confirm improved colonic dilatation and no findings suggesting persistent severe obstruction or perforation. CT was performed or reviewed when clinically indicated. Length of hospital stay was calculated from admission to discharge or in‐hospital death.

Among patients with clinical success after endoscopic management, the in‐hospital course was evaluated using time to resumption of oral intake and length of hospital stay. Endoscopic mucosal findings at the index procedure (normal mucosa, erythema, or necrosis) were recorded as procedural findings.

The recurrence outcome was cumulative SV recurrence within 1 year after index endoscopic management. Recurrence was defined as recurrent obstructive symptoms with imaging findings suggestive of SV requiring medical attention and/or intervention. Cumulative recurrence was evaluated using time‐to‐event methods through 365 days. Patients were censored at elective surgery before recurrence or at the last available follow‐up within 365 days.

Elective surgery was planned after improvement from the index episode to prevent recurrence. Indications were individualized by attending physicians and surgeons according to postprocedural stability, recurrence risk, age, performance status, comorbidities, activities of daily living, cardiopulmonary reserve, surgical tolerance, and patient/family preference. Because recommendations were not standardized, patients not undergoing elective surgery could not be reliably classified as “not recommended” or “recommended but declined.”

### 2.7 Statistical Analysis

Continuous variables are presented as mean ± standard deviation or median (interquartile range [IQR]), as appropriate. Between‐group comparisons were performed using Welch's t test for approximately normally distributed continuous variables and the Mann–Whitney U test for skewed variables. Categorical variables are presented as counts and percentages and were compared using Fisher's exact test. The distribution of endoscopic findings was compared using an exact test for contingency tables. Cumulative recurrence was estimated using the Kaplan–Meier method and compared using the log‐rank test. All tests were two‐sided, and *p* < 0.05 was considered statistically significant. No adjustment for multiple comparisons was performed. Patients who underwent elective surgery before recurrence were censored at the time of surgery, and those without recurrence were censored at the last available follow‐up within 365 days.

## 3 Results

### 3.1 Patient Enrollment and Endoscopic Treatment Outcomes

**FIGURE 1:**
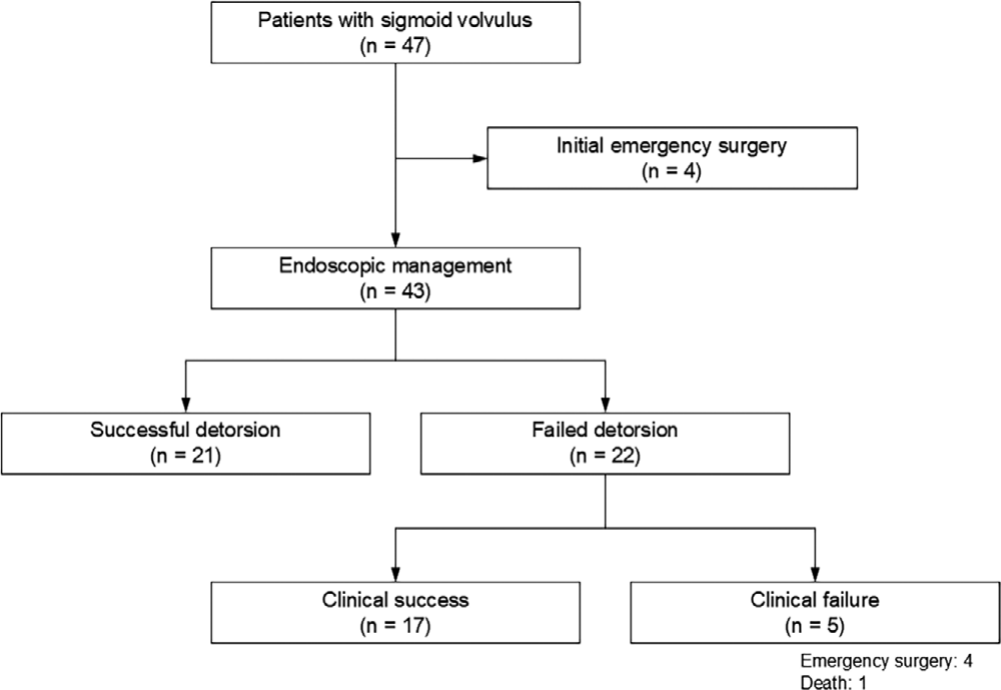
Patient flow chart. Among 47 patients with sigmoid volvulus (SV), 4 underwent initial emergency surgery and 43 underwent endoscopic management. Successful endoscopic detorsion was achieved in 21 patients, whereas detorsion failed in 22 patients. Among patients with failed detorsion, 17 achieved clinical success after decompression, whereas five had clinical failure, including four who underwent emergency surgery and one who died.

During the study period, 47 patients with first‐episode SV were identified. Four patients underwent initial emergency surgery, and 43 underwent endoscopic management (Figure 1). Successful endoscopic detorsion was achieved in 21 of 43 patients (48.8%). Among the remaining 22 patients with failed detorsion, 17 achieved clinical success after decompression, whereas 5 had clinical failure, including four who underwent emergency surgery and one who died the following day without surgery because of poor general condition. The overall clinical success rate of endoscopic management was 88.4% (38/43).

For analyses of baseline characteristics and short‐term outcomes, the successful detorsion group (*n* = 21) was compared with the failed detorsion group (*n* = 22). For analyses of the in‐hospital course and recurrence after clinical improvement, the successful detorsion group (*n* = 21) was compared with the successful decompression group (*n* = 17).

### 3.2 Baseline Characteristics

Baseline characteristics are summarized in Tables 1 and 2. Age, sex, BMI, performance status, and comorbidities were similar between the successful and failed detorsion groups. At presentation, body temperature, abdominal distension, and spontaneous abdominal pain did not differ significantly between groups.

**TABLE 1:** Baseline demographic and comorbidity characteristics of patients managed endoscopically.

Variable	Successful detorsion (*n* = 21)	Failed detorsion (*n* = 22)	*p*‐Value
Age, years (mean ± SD)	75.9 ± 12.6	74.9 ± 12.1	0.783
Male sex, *n* (%)	16 (76%)	17 (77%)	1.000
BMI, kg/m^2^ (mean ± SD)	21.7 ± 3.4	20.3 ± 4.0	0.250
Performance status, ECOG scale (mean ± SD)	1.4 ± 1.6	1.8 ± 1.6	0.419
Dementia, *n* (%)	3 (14%)	5 (23%)	0.750
Cerebrovascular disease, *n* (%)	6 (29%)	6 (27%)	0.924
Parkinsonism, *n* (%)	2 (10%)	4 (18%)	0.705
Psychiatric disorders, *n* (%)	5 (24%)	3 (14%)	0.642
Cardiac disease, *n* (%)	11 (52%)	12 (55%)	0.887
Respiratory disease, *n* (%)	5 (24%)	5 (23%)	1.000
Diabetes mellitus, *n* (%)	4 (19%)	6 (27%)	0.782
Prior abdominal surgery, *n* (%)	7 (33%)	6 (27%)	0.665
Laxative use, *n* (%)	14 (67%)	15 (68%)	0.916
Residence in a long‐term care facility or chronic care hospital, *n* (%)	8 (38%)	9 (41%)	0.850

Abbreviations: BMI: body mass index; ECOG: Eastern Cooperative Oncology Group; SD: standard deviation.

**TABLE 2:** Baseline clinical presentation and laboratory data of patients managed endoscopically.

Variable	Successful detorsion (*n* = 21)	Failed detorsion (*n* = 22)	*p*‐Value
Body temperature, °C (mean ± SD)	36.7 ± 0.5	36.7 ± 0.5	0.916
SpO_2_ on room air, % (mean ± SD)	96.8 ± 2.2	95.2 ± 2.7	0.038
Abdominal distension, *n* (%)	21 (100%)	22 (100%)	1.000
Spontaneous abdominal pain, *n* (%)	13 (62%)	13 (59%)	0.850
Albumin, g/dL (mean ± SD)	3.8 ± 0.5	3.8 ± 0.5	0.883
LDH, U/L (mean ± SD)	236.0 ± 82.9	223.7 ± 65.8	0.592
eGFR, mL/min/1.73 m^2^ (mean ± SD)	65.8 ± 22.5	59.5 ± 27.8	0.421
CK, U/L (mean ± SD)	164.2 ± 170.5	161.1 ± 214.5	0.961
HbA1c, % (mean ± SD)	5.8 ± 0.4	6.1 ± 1.4	0.335
CRP, mg/dL (mean ± SD)	0.6 ± 0.9	3.5 ± 7.0	0.072
WBC, /µL (mean ± SD)	7750 ± 3455	9480 ± 4334	0.155
Platelets, ×10^4^/µL (mean ± SD)	19.5 ± 5.4	23.2 ± 10.2	0.140
PT‐INR (mean ± SD)	1.0 ± 0.1	1.0 ± 0.1	0.571
Lactate, mmol/L (mean ± SD)	1.5 ± 0.6	3.0 ± 2.3	0.017

Abbreviations: CK, creatine kinase; CRP, C‐reactive protein; eGFR, estimated glomerular filtration rate; HbA1c, hemoglobin A1c; LDH, lactate dehydrogenase; PT‐INR, prothrombin time–international normalized ratio; SD, standard deviation; SpO_2_, peripheral oxygen saturation; WBC, white blood cell count.

Among laboratory and physiologic variables at admission, SpO2 was higher in the successful detorsion group than in the failed detorsion group (96.8 ± 2.2% vs. 95.2 ± 2.7%, *p* = 0.038), whereas lactate levels were lower (1.5 ± 0.6 vs. 3.0 ± 2.3 mmol/L, *p* = 0.017). Other clinical and laboratory variables, including albumin, inflammatory markers, and renal function, did not differ significantly between groups.

### 3.3 Endoscopic Findings

Endoscopic findings at the index colonoscopy are summarized in Table 3. Normal mucosa was observed in 16 of 21 patients (76%) in the successful detorsion group and 12 of 22 (55%) in the failed detorsion group, whereas mucosal necrosis was observed only in the failed detorsion group (four of 22, 18%). The overall distribution of endoscopic findings did not differ significantly between groups (overall *p* = 0.098). Among the four patients with endoscopic mucosal necrosis, three underwent urgent surgery, whereas one patient died the following day before surgery could be performed. Additionally, one patient without endoscopic mucosal necrosis underwent urgent surgery because of clinical failure after endoscopic management.

**TABLE 3:** Endoscopic findings at index colonoscopy.

Variable	Successful detorsion (*n* = 21)	Failed detorsion (*n* = 22)	Overall *p*‐Value
Normal mucosa, *n* (%)	16 (76%)	12 (55%)	0.098
Erythema, *n* (%)	5 (24%)	6 (27%)	
Necrosis, *n* (%)	0 (0%)	4 (18%)	

*p*‐Value was calculated for the overall distribution of endoscopic findings between the groups using an exact test for contingency tables.

### 3.4 Short‐term Outcomes

Short‐term outcomes are summarized in Table 4. Clinical success was achieved in all patients in the successful detorsion group and in 17 of 22 patients (77%) in the failed detorsion group (100% vs. 77%, *p* = 0.065). Emergency surgery was required in 0 of 21 patients (0%) in the successful detorsion group and in four of 22 patients (18%) in the failed detorsion group (*p* = 0.127). No patients in the successful detorsion group died within 30 days, whereas one patient (5%) in the failed detorsion group died within 30 days (*p* = 1.000). Table 5 summarizes the nine patients who underwent initial emergency surgery, urgent surgery after endoscopy, or died during the index hospitalization, including lactate, CT ascites, endoscopic findings, and time from endoscopy to surgery/death.

**TABLE 4:** Short‐term outcomes of endoscopic management.

Outcome	Successful detorsion (*n* = 21)	Failed detorsion (*n* = 22)	*p*‐Value
Clinical success, *n* (%)	21 (100%)	17 (77%)	0.065
Emergency surgery, *n* (%)	0 (0%)	4 (18%)	0.127
30‐day mortality, *n* (%)	0 (0%)	1 (5%)	1.000
Length of hospital stay, days, median (IQR)	7 (5–10)	13 (6–25)	0.071

IQR: interquartile range.

**TABLE 5:** Clinical summary of patients who underwent emergency or urgent surgery or those who died during the index hospitalization.

Patient No.	Management pathway	Age/Sex	ECOG PS score	Lactate levels at admission, mmol/L	Ascites confirmed via CT	Endoscopic mucosal finding	Endoscopic evidence of mucosal necrosis	Time from endoscopy to surgery/death	Hospital stay, days
1	Initial emergency surgery without endoscopy	73/Male	0	5.7	Yes	Not assessed	Not assessed	Not applicable	12
2	Initial emergency surgery without endoscopy	77/Female	4	1.8	Yes	Not assessed	Not assessed	Not applicable	27
3	Initial emergency surgery without endoscopy	83/Male	0	3.6	Yes	Not assessed	Not assessed	Not applicable	34
4	Initial emergency surgery without endoscopy	53/Male	3	7.3	Yes	Not assessed	Not assessed	Not applicable	70
5	Urgent surgery after endoscopy	83/Male	3	3.4	Yes	Necrosis	Yes	30 min	14
6	Urgent surgery after endoscopy	76/Male	2	8.1	Yes	Necrosis	Yes	1 h	25
7	Urgent surgery after endoscopy	69/Male	2	2.1	Yes	Erythema	No	7.5 h	79
8	Urgent surgery after endoscopy	90/Male	2	3.7	Yes	Necrosis	Yes	24 h	38
9	Death after endoscopy without surgery	89/Male	3	5.0	Yes	Necrosis	Yes	15 h	2

CT, computed tomography; ECOG PS, Eastern Cooperative Oncology Group Performance Status.

Ascites was assessed via a CT performed at presentation.

Endoscopic mucosal findings were marked as “Not assessed” in patients who proceeded directly to initial emergency surgery without endoscopy.

“Not applicable” indicates that endoscopy was not performed before surgery.

The median length of hospital stay tended to be shorter in the successful detorsion group than in the failed detorsion group (7 [IQR 5–10] vs. 13 [IQR 6–25] days, *p* = 0.071).

Among patients with clinical success after endoscopic management, the median time to resumption of oral intake was shorter in the successful detorsion group than in the successful decompression group (1.5 [IQR 1–2] vs. 3 [IQR 2–5] days, *p* = 0.004), whereas the median length of hospital stay did not differ significantly between groups (7 [IQR 5–10] vs. 9 [IQR 6–16] days, *p* = 0.185).

### 3.5 Recurrence Among Patients With Clinical Success

**FIGURE 2:**
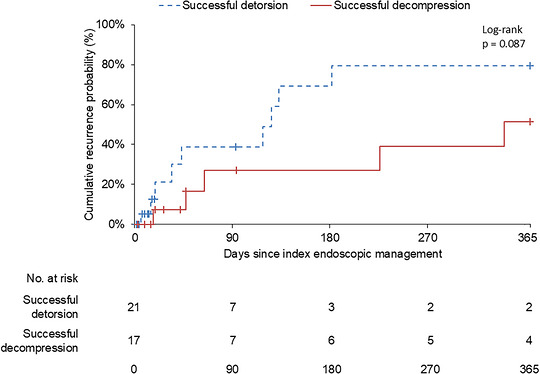
Cumulative recurrence within 1 year among patients with clinical success after endoscopic management. The successful detorsion group (*n* = 21) and the successful decompression group (*n* = 17) were compared using the Kaplan–Meier method. The 1‐year cumulative recurrence rate tended to be higher in the successful detorsion group than in the successful decompression group (79.6% vs. 51.3%, log‐rank *p* = 0.087). Patients were censored at the time of elective surgery or at the last available follow‐up within 365 days. Numbers at risk are shown below the plot.

Cumulative recurrence within 1 year among patients with clinical success after endoscopic management is shown in Figure 2. The Kaplan–Meier estimate of 1‐year cumulative recurrence was numerically higher in the successful detorsion group than in the successful decompression group, although the difference was not statistically significant (79.6% vs. 51.3%, log‐rank *p* = 0.087).

Within 1 year, loss to follow‐up occurred in both groups (nine of 21 in the detorsion group and four of 17 in the decompression group), whereas elective surgery before recurrence was performed in one of 21 patients in the detorsion group and four of 17 patients in the decompression group.

## 4 Discussion

This single‐center retrospective cohort study had three main findings. First, successful endoscopic detorsion was achieved in approximately half of endoscopically managed first‐episode SV patients, whereas overall clinical success was high. Second, failed detorsion was associated with less favorable short‐term outcomes, including a lower clinical success and longer hospital stay. Third, among patients with clinical success, oral intake resumed earlier after successful detorsion, whereas 1‐year cumulative recurrence tended to be higher than after successful decompression.

Urgent endoscopic management is widely accepted as first‐line treatment for uncomplicated SV [2, 5, 6]. However, evidence regarding the clinical course after failed detorsion remains limited. Nakamatsu et al. reported no significant differences in recurrence‐related outcomes between successful detorsion and decompression after failed detorsion in uncomplicated SV [9]. The present findings extend these observations by separately evaluating short‐term outcomes in the overall endoscopic cohort and subsequent outcomes among patients who achieved clinical success after decompression.

The detorsion success rate in this study was 48.8%, which is lower than that reported in some previous series [2, 4, 8]. This likely reflects differences in patient severity, procedural difficulty, operator decision‐making, and definitions of technical success. Nevertheless, although technical success was achieved in only about half of the cohort, overall clinical success was observed in 88.4% of patients. This distinction is clinically relevant. In routine practice, failed detorsion does not necessarily indicate immediate clinical failure; a substantial proportion of patients may improve after decompression alone and avoid immediate surgery.

At baseline, the failed detorsion group had lower SpO_2_ and higher lactate levels, and mucosal necrosis was observed only in this group, suggesting more severe physiologic compromise at presentation. These findings raise the possibility of confounding by indication, whereby endoscopists may have been more likely to terminate detorsion attempts early in patients considered to be at higher procedural risk or with more severe disease. The short‐term outcomes were consistent with this interpretation: clinical success was less frequent, and hospital stay was longer in the failed detorsion group. However, when the analysis was restricted to patients who ultimately achieved clinical success, the difference in hospital stay was no longer observed, whereas time to resumption of oral intake remained shorter in the successful detorsion group. These findings suggest that complete detorsion may facilitate faster early recovery, whereas among patients who show improvement after decompression, the subsequent in‐hospital course may be comparable. The individual summary of patients who underwent emergency surgery or died during the index hospitalization further illustrates the clinical context of these severe outcomes, including lactate levels, CT findings of ascites, and endoscopic evidence of mucosal necrosis when assessable (Table 5). Because this summary was descriptive and involved only nine patients, the findings should not be interpreted as independent predictors of emergency surgery or mortality.

Recurrence remains a major concern after endoscopic treatment for SV [2, 4, 7, 8]. The numerically higher 1‐year recurrence after successful detorsion should not be interpreted as evidence favoring decompression. This exploratory finding may reflect selection and censoring effects, including exclusion of clinical failures after decompression and more frequent elective surgery before recurrence in the decompression group, as well as anatomical differences such as sigmoid redundancy. Therefore, the recurrence analysis is hypothesis‐generating.

From a clinical perspective, endoscopic detorsion should remain the preferred therapeutic objective in uncomplicated SV. However, when complete detorsion cannot be safely achieved, decompression alone may serve as a feasible salvage option in selected patients who subsequently demonstrate clinical improvement. Patients who do not resume oral intake or otherwise fail to show improvement after decompression should undergo prompt surgical evaluation.

This study has some limitations. First, it was a retrospective single‐center study, which may limit generalizability. Second, the sample size was small, reducing statistical power, particularly for recurrence analyses. Third, the decision to continue or abandon detorsion and proceed with decompression alone was at the discretion of the endoscopist and was not standardized. Fourth, recurrence analysis was restricted to patients who achieved clinical success, introducing selection bias. Finally, elective surgery before recurrence and variable follow‐up may have influenced recurrence estimates, and competing‐risk analysis was not performed. Larger multicenter studies with standardized procedural criteria and follow‐up protocols are needed to clarify which patients benefit most from decompression after failed detorsion.

In this single‐center retrospective cohort, successful endoscopic detorsion was achieved in approximately half of patients with SV, whereas many patients with failed detorsion achieved clinical success after decompression alone. Among patients with clinical success, time to resumption of oral intake was shorter in the successful detorsion group, whereas the 1‐year cumulative recurrence rate tended to be higher than that in the successful decompression group; however, this recurrence finding should be interpreted cautiously because of selection and censoring bias. These findings suggest that decompression alone may be a feasible salvage strategy after failed endoscopic detorsion in selected patients who achieve clinical improvement, provided that close postprocedural monitoring and timely surgical escalation are ensured for those who do not improve.

## Author Contributions

**Hidehiro Kamezaki**: conceptualization; methodology; formal analysis, investigation; writing – original draft; writing – review and editing; supervision. **Arisa Kato**: data curation; visualization; writing – review & editing. **Nana Yamada**, **Terunao Iwanaga**, **Takafumi Sakuma**, **Koji Takahashi**, **Junichi Senoo**, **Sadahisa Ogasawara**, **Tomoaki Matsumura**, **Jun Kato**: writing – review and editing; data interpretation.

## Funding

The authors have nothing to report.

## Ethics Statement

This study was conducted in accordance with the Declaration of Helsinki and was approved by the institutional ethics committee of Eastern Chiba Medical Center (approval no. 299).

## Consent

Because of the retrospective design, the requirement for written informed consent was waived, and an opt‐out approach was used.

## Conflicts of Interest

The authors declare no conflicts of interest.

## Data Availability

The data that support the findings of this study are available on request from the corresponding author. The data are not publicly available due to privacy or ethical restrictions.
